# Transcriptome Analysis and Discovery of Genes Involved in Immune Pathways from Coelomocytes of Sea Cucumber (*Apostichopus japonicus*) after *Vibrio splendidus* Challenge

**DOI:** 10.3390/ijms160716347

**Published:** 2015-07-17

**Authors:** Qiong Gao, Meijie Liao, Yingeng Wang, Bin Li, Zheng Zhang, Xiaojun Rong, Guiping Chen, Lan Wang

**Affiliations:** 1 Fisheries College, Ocean University of China, Qingdao 266100, China; E-Mail: gaohaijun1989@163.com; 2 Key Laboratory of Sustainable Development of Marine Fisheries, Ministry of Agriculture, Yellow Sea Fisheries Research Institute, Chinese Academy of Fishery Sciences, Qingdao 266071, China; E-Mails: liaomj@ysfri.ac.cn (M.L.); libin@ysfri.ac.cn (B.L.); zhangzheng@ysfri.ac.cn (Z.Z.); rongxj@ysfri.ac.cn (X.R.); chengp@ysfri.ac.cn (G.C.); wanglan_0829@163.com (L.W.)

**Keywords:** sea cucumber (*Apostichopus japonicus*), *Vibrio splendidus*, transcriptome sequencing, differentially expressed genes, bacteria-resistant gene, bacteria-susceptible gene

## Abstract

*Vibrio splendidus* is identified as one of the major pathogenic factors for the skin ulceration syndrome in sea cucumber (*Apostichopus japonicus*), which has vastly limited the development of the sea cucumber culture industry. In order to screen the immune genes involving *Vibrio splendidus* challenge in sea cucumber and explore the molecular mechanism of this process, the related transcriptome and gene expression profiling of resistant and susceptible biotypes of sea cucumber with *Vibrio splendidus* challenge were collected for analysis. A total of 319,455,942 trimmed reads were obtained, which were assembled into 186,658 contigs. After that, 89,891 representative contigs (without isoform) were clustered. The analysis of the gene expression profiling identified 358 differentially expression genes (DEGs) in the bacterial-resistant group, and 102 DEGs in the bacterial-susceptible group, compared with that in control group. According to the reported references and annotation information from BLAST, GO and KEGG, 30 putative bacterial-resistant genes and 19 putative bacterial-susceptible genes were identified from DEGs. The qRT-PCR results were consistent with the RNA-Seq results. Furthermore, many DGEs were involved in immune signaling related pathways, such as Endocytosis, Lysosome, MAPK, Chemokine and the ERBB signaling pathway.

## 1. Introduction

Currently, the sea cucumber (*Apostichopus japonicus*) has become one of the most important aquaculture species in China, achieving valuable profits [1]. However, many diseases occurred along with the rapid expansion and intensification of farming, causing serious economic losses and disrupting the sustainable development of this industry [2]. Skin ulceration syndrome with clinical signs of anorexia, shaking head, mouth timidity, viscera ejection and skin ulceration, is the most serious disease and is highly infectious and lethal to the species. Etiological studies [3,4,5] indicated that a bacterial species (*Vibrio*/*Pseudoalteromonas*/*Aeromonas* spp.), a parasite (Parasitic nematode) and virus (a spherical virus) are the major pathogens for this serious disease, including *Vibrio splendidus* [6].

Like other invertebrates, sea cucumber lacks adaptive immunity and relies solely on innate immunity, which is composed of cellular responses such as phagocytosis and encapsulation, as well as humoral immunity that produces immune-related factors [7,8]. Coelomocytes function as the major sites for the elimination of pathogens [9,10]. Moreover, there are plentiful hydrolases in coelomic fluid such as lysozyme (LSZ), phenoloxidase activity (PO), total nitric oxide synthase (T-NOS), superoxide dismutase (SOD), and alkaline phosphatase (AKP), which could hydrolyze foreign pathogens [11,12]. In recent years, various studies have been conducted to investigate the immunity of sea cucumber, trying to identify the immune factors, clone immune-related genes, and obtain immune-related expressed sequence tags (EST) [13,14,15,16,17,18]. In addition, the studies of different microRNAs and proteins between healthy and natural skin ulceration syndrome *Apostichopus japonicus* have also been performed [19,20,21]. Nevertheless, the responsive mechanism of sea cucumber to pathogenic bacteria remains unclear.

In the present study, a mid-sensitive full-sib family was chosen for artificial-challenge experiment using *Vibrio splendidus.* The sea cucumbers were divided into a disease-resistant group and susceptibility group according to a series of symptoms of skin ulcer syndrome [4]. The transcriptome and expression profile of bacterial-resistant and bacterial-susceptible sea cucumber juveniles’ coelomocytes were analyzed using the Illumina sequencing method and bioinformatics analysis. Putative disease-resistant genes and susceptibility genes of sea cucumber were also screened. Additionally, gene-associated markers were screened for potential genetic research.

## 2. Results and Discussion

### 2.1. Illumina Sequencing and Assembly

**Figure 1:**
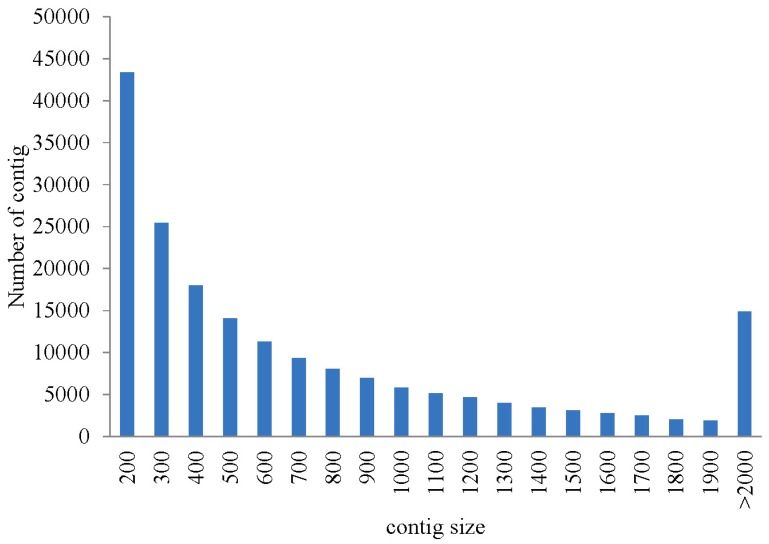
The length distribution of assembled contigs in the sequenced cDNA library.

**Figure 2:**
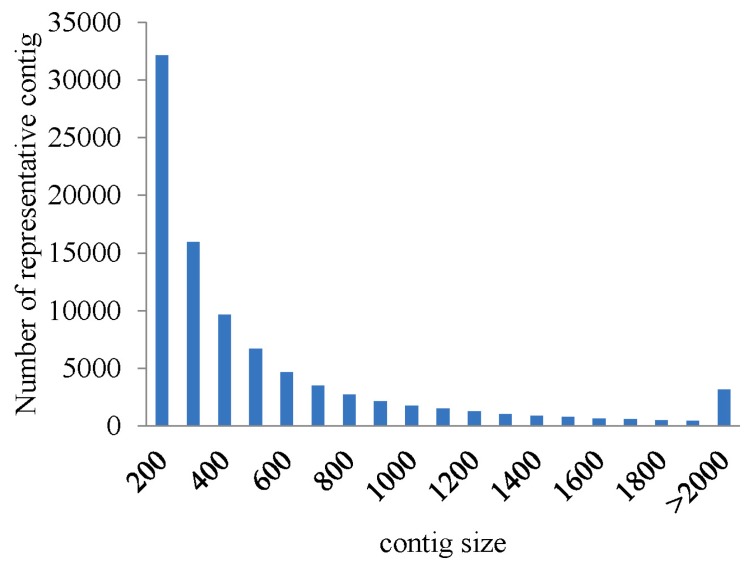
The length distribution of representative contigs in the sequenced cDNA library.

To obtain an overview of the sea cucumber coelomocytes transcriptome, a cDNA library was generated from an equal mixture of RNA from nine individuals of the three groups (control group, bacterial-resistant group, and bacterial-susceptible group) using Illumina Hiseq2500 platform. After cleaning and quality checks, approximately 319 million (319,455,942) trimmed reads from 320 million (320,106,172) raw reads were generated using deep sequencing. Assembling analysis obtained 186,658 contigs with a median of contigs (N50) length of 1245, and 89,891 representative contigs (without isoform) with a N50 length of 791 using the 25-mer parameter in Trinity [22,23] (Table 1). Length statistics of assembled contigs and representative contigs are displayed in Figure 1 and Figure 2. The transcriptome database was next used as a source for the large set of functional genes (disease-resistant genes and susceptibility genes). The abundant data could also be a reference for further study including molecular markers and the genome of sea cucumber.

**Table 1:** Summary statistics of assembled transcriptome length for *Apostichopus japonicus* coelomocytes.

Assembled Transcriptome	All	Min	Median	Mean	N50	Max	Total
contigs	186,658	201	543	832	1245	15,051	155,375,852
representative contigs (without isoform)	89,891	201	376	593	791	15,051	53,310,798

N50, contig length—weighted median.

### 2.2. Gene Annotation

Representative contigs were first annotated by BLAST to protein databases nr, Swiss-prot, Pfam, KEGG and COG separately and then annotated to nucleotide databases Nt with an *E*-value cut-off of 10^−5^. The percent values of the representative contigs to these databases are listed in Table 2. Altogether, 20,060 (22.32%) had at least one significant match to these databases (Table 2).

**Table 2:** Result of functional annotation of the assembled representative contigs to the databases.

No. of Representative Contigs	Swiss-Prot	Nr	Pfam	KEGG	COG
89,891	13,955	19,777	14,398	18,887	13,126
Percentage	15.52%	22.00%	16.02%	21.01%	14.60%

#### 2.2.1. Nr Annotation

**Figure 3:**
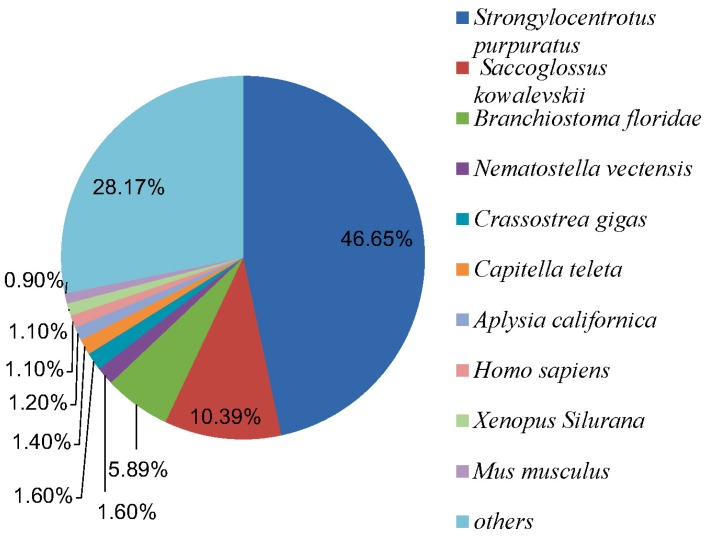
Species distribution of the BLAST matches of the transcriptome representative contigs.

Among the annotated representative contigs to the Nr database, 6724 (46.7%) representative contigs were matched to *Strongylocentrotus* *purpuratus*, 1497 (10.4%) to *Saccoglossus* *kowalevskii*, 849 (5.9%) to *Branchiostoma floridae*, 230 (1.6%) to *Nematostella vectensis*, 230 (1.6%) to *Crassostrea gigas*, 202 (1.4%) to *Capitella teleta*, 173 (1.2%) to *Aplysiacali fornica*, 158 (1.1%) to *Homo sapiens*, 158 (1.1%) to *Xenopus silurana*, 130 (0.9%) to *Mus musculus* and 4047 (28.2%) to other species (Figure 3).

#### 2.2.2. GO Annotation

**Figure 4:**
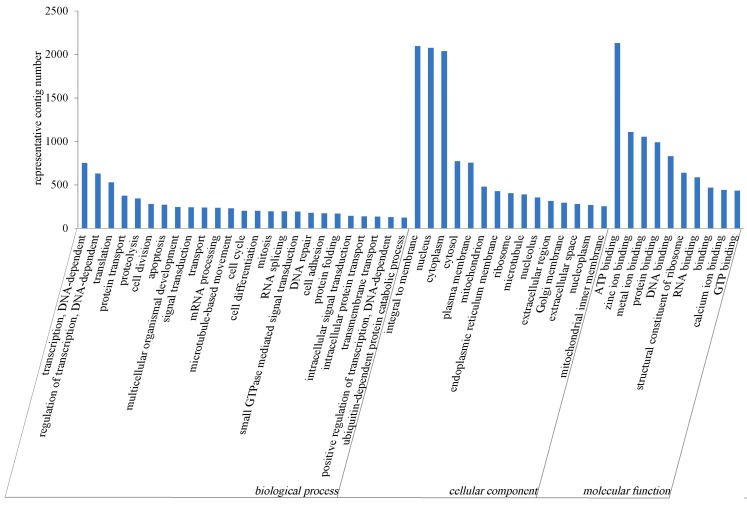
Classification of the gene ontology (GO) for the sea cucumber coelomocytes transcriptome representative contigs.

Gene Ontology (GO) [24] analysis was carried out, and for the three major functional categories: biological process, cellular component and molecular function, there were 9574, 11,078 and 10,994 representative contigs, respectively (Figure 4). For biological process, genes involved in transcription and DNA-dependent (752) was highest represented, followed by regulation of transcription DNA-dependent (630), and translation (528). Regarding cellular component, the top three categories were integral to membrane (2095), nucleus (2075) and cytoplasm (2037). For molecular function, ATP binding was the most represented GO term, followed by zinc ion binding.

#### 2.2.3. COG Annotation

**Figure 5:**
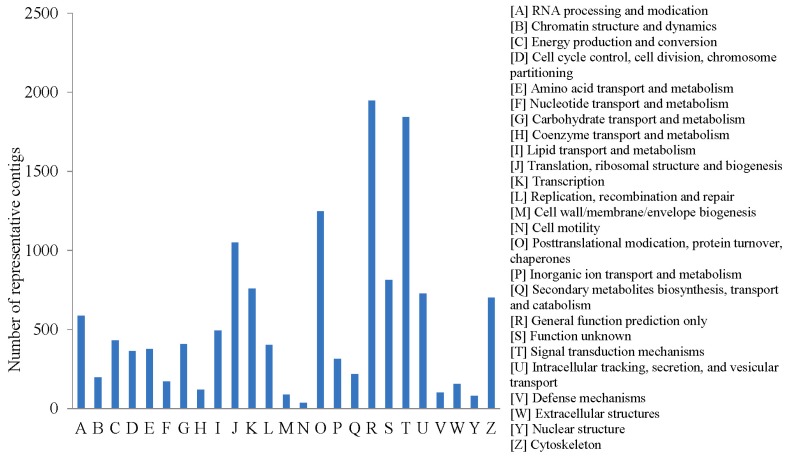
Clusters of Orthologous Groups (COG) classification of the sea cucumber coelomocytes transcriptome representative contigs.

The COG database was used to classify orthologous gene products. 13,126 representative contigs were allocated to 25 COG classifications (Figure 5). Among them, “general function prediction only” (1946, 14.83%) and “signal transduction mechanisms” (1844, 14.05%) was the largest group, which indicated multiple genes were involved in signaling pathways after *Vibrio splendidus* infection.

#### 2.2.4. KEGG Annotation

**Figure 6:**
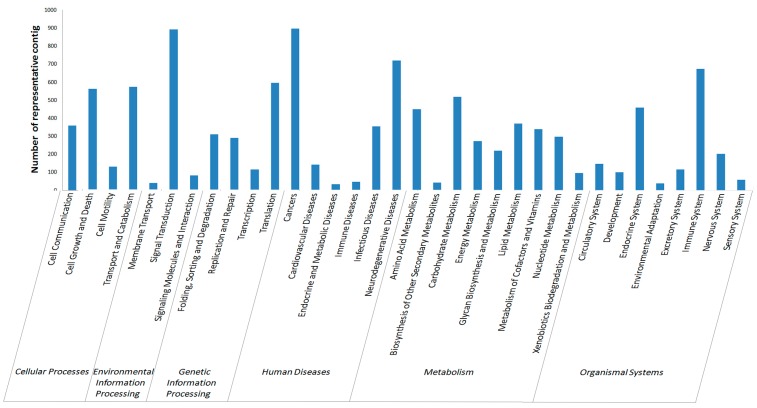
KEGG classification of the sea cucumber coelomocytes transcriptome representative contigs.

KEGG was used as a powerful tool to analyze biological metabolism and study metabolism networks. In all, 18,887 representative contigs were consequently classified into specific pathways (Figure 6), among which maximum members fell into “metabolism” (2346) and “human diseases” (2151), followed by “organism system” (1779), “cellular processes” (1625) and “genetic information processing” (1308), while the least amount of members were assigned to “environmental information processing” (1009). The highest number of genes were involved in signal transduction and immune system in KEGG, indicating many genes could respond to *Vibrio* *splendidus* challenge.

### 2.3. Single Nucleotide Polymorphism (SNP) and Simple Sequence Repeat (SSR) Detecting

The transcriptome is also an important EST resource for rapid and effective mining of genetic markers, such as SNP and SSR [25]. The molecular markers have also been widely used in identifying functional genes, genetic breeding, genome mapping, and cloning genes.

In total, 149,745 high-quality SNPs were detected using Bowtie and the SAMTOOLS software. The dominant type of variation was transition (86,345, 57.67%), followed by transversion (63,391, 42.33%). The most common transition type was A→G and C→T (Table 3).

**Table 3:** Summary of SNP identified from the sea cucumber coelomocytes transcriptome.

SNP Type	NO. of SNP
Transition	86,354
A-G	41,651
C-T	44,703
Transversion	63,391
A-C	16,243
A-T	21,687
C-G	10,327
G-T	15,134
Total	149,745

**Figure 7:**
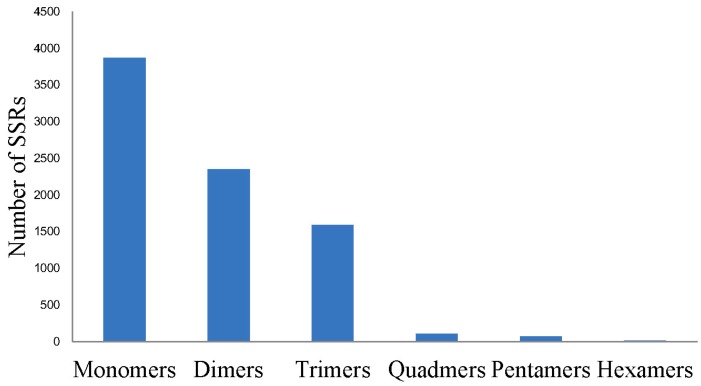
Summary of simple sequence repeat (SSR) identified from the sea cucumber coelomocytes transcriptome.

In addition, 8009 SSRs (simple sequence repeats) were identified from the assembled sequences. The most abundant repeat motifs were mono-nucleotides (3869), which accounted for 48.31% of all SSRs, followed by dinucleotides (2350, 29.34%), trinucleotides (1591, 19.87%), tetranucleotides (111, 1.39%), pentanucleotides (70, 0.87%), and hexanucleotides (18, 0.22%) (Figure 7).

### 2.4. Construction of Digital Expression Profiling for Differentially Expressed Genes

**Figure 8:**
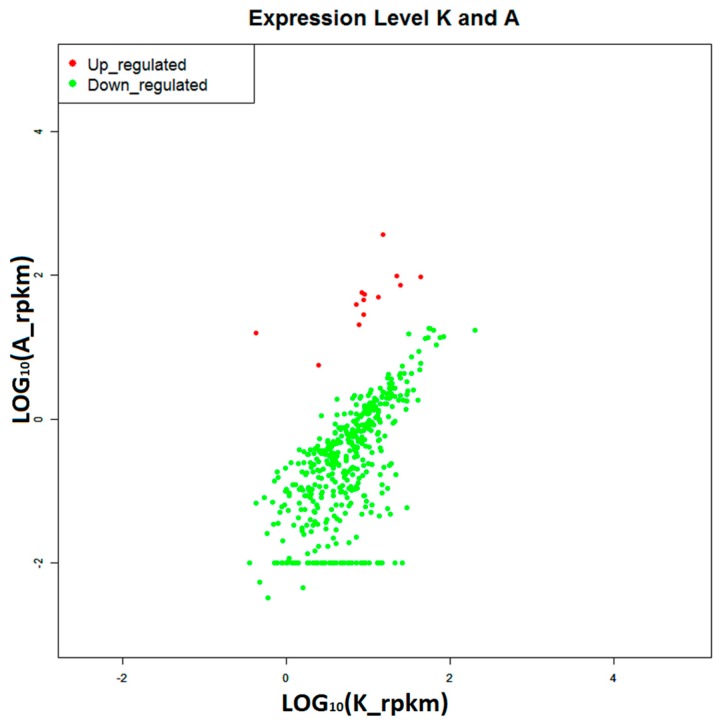
Differentially expressed genes from disease-resistant group (A), comparing with control group (K). Red points represent 13 up-regulated genes, and green points represent 345 down-regulated genes.

**Figure 9:**
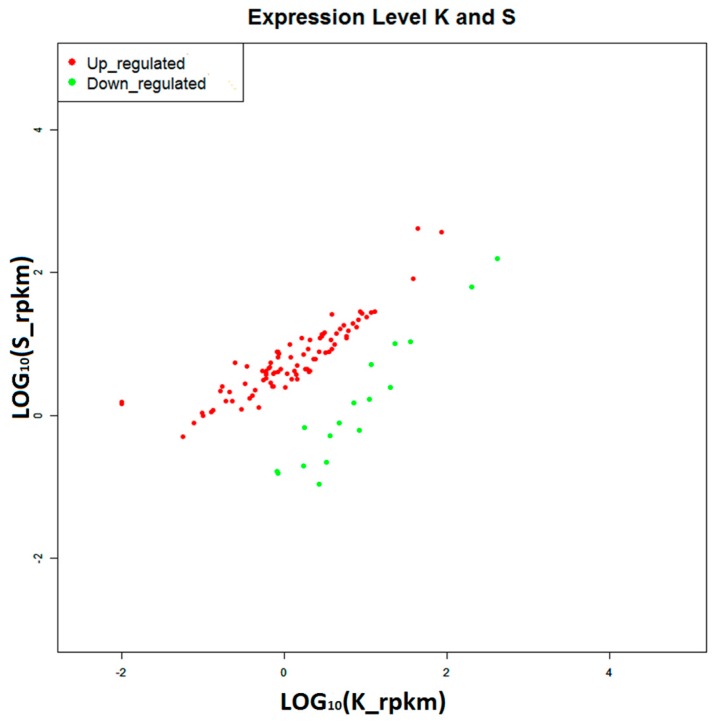
Differentially expressed genes from susceptibility group (S) comparing with control group (K). Red points represent 86 up-regulated genes, green point represents 16 down-regulated genes.

The digital gene expression profiling (DGE) is a rapid and efficient approach for gene expression analysis [26,27]. Many significantly differentially expressed genes (DEGs) were acquired by comparing the gene expressions in disease-resistant or susceptibility group (A or S, respectively) with the control group (K), under the criteria of *p*-value ≤ 0.01 and |log_2_ fold-change (FC)| ≥ 1 (FDR ≤ 0.05). As a result, we obtained 358 DEGs in the disease-resistant group (13 up-regulated and 345 down-regulated) (Figure 8) and 102 DEGs in the susceptibility group (86 up-regulated and 16 down-regulated) (Figure 9).

### 2.5. Selecting Disease-Resistant and Susceptibility Genes

The information of genes involved in immune response of other species were collected, which were used in combination of the GO, KEGG, NCBI annotation for the identification of potential bacterial-resistant genes, and 30 genes were identified (listed in Table 4 and Table 5). Some of these 30 genes had been reported in sea cucumber, such as heat shock protein70 (HSP70-like), which is a member of the heat shock protein family, stimulates the innate immune response [28,29] and plays crucial roles in environmental stress tolerance and adaptation in sea cucumber [30,31]. HSP70-like expression was significantly up-regulated after *Vibrio splendidus* infection, which is consistent with the result of LPS challenge [32].

In addition, many disease-resistant genes that had not been previously linked to the immune response, including heat-responsive protein 12 (Hrp12-like), serine/threonine-protein kinase RIO3 (RIOK3-like) and Interferon-induced very large GTPase 1 (Gvin1-like) were involved in the immune response. However, these genes had been investigated in other species. For instance, Hrp12 has significant similarity to Hsp70 [33] and Hrp12-like may also play an important role in protein transport, protein folding and cell signaling. RIOK3 is a novel regulator of the antiviral type I interferon pathway and plays a crucial role in the antiviral type I interferon pathway. However, type I interferon is involved in the innate immune response which functions as the first line of defense and limits infectious pathogens directly [34], therefore we predicted that RIOK3-like is related closely to innate immunity. Mitogen-activated protein kinase kinase 6 (MP2K6), a member of the MAPK family which are signal transduction mediators that have been implicated in cell survival and death [35], is also activated during engagement of the Type I IFN receptor and plays important roles in Type I IFN signaling and generation of IFN responses [36]. Gvin1 that contributes to the cellular response to both type I and type II IFNs could lead to cell-autonomous resistance against various pathogens [37].

Nineteen potential susceptibility genes (Table 4 and Table 6) were identified from the DEGs, including Rho GTPase-activating protein 39 (ARHGAP39-like), scavenger receptor cysteine-rich protein type 12 precursor (DMBT1-like), and nuclear factor NF-κB p105 subunit (NFkB-like). The Rho GTPase-activating proteins (RhoGAPs) are one of the major classes of regulators of Rho GTPase which are important in cell cytoskeletal organization, membrane trafficking, transcriptional regulation, cell growth and differentiation, neuronal morphogenesis, and endocytosis [38,39]. Thus, ARHGAP39-like may be related with the immune response. Members of the scavenger receptor cysteine-rich (SRCR) superfamily have diverse functions, including pathogen recognition and immune-regulation [40], and we inferred that DMBT1-like might be also involved in the immune response.

**Table 4:** A subset of candidate *Vibrio splendidus*-resistant and susceptibility genes that are involved in the immune signaling pathway.

Coding Number	Contig ID	Gene Name	Predict Function	Regulation	Log_2_ FC	Accession Number in Nr Database	Identities (%)
**Chemokine signaling pathway**							
A1	comp76725_c0_seq6	FOXO1-like	Fork head box protein (*Strongylocentrotus purpuratus*)	Down	−2.23	XP_790591.3	78
A2	comp78415_c0_seq14	ADCY2-like	Adenylatecyclase type 2 (*Strongylocentrotus purpuratus*)	Down	−5.91	XP_780688.3	72
A3	comp79708_c0_seq1	STAT5B-like	Signal transducer and activator of transcription 5B (*Strongylocentrotus purpuratus*)	Down	−3.81	XP_003723422.1	70
S1	comp79328_c1_seq13	NFKB-like	Nuclear factor NF-κB p105 subunit (*Apostichopus japonicus*)	Up	1.8	AEP33644.1	68
S2	comp74502_c1_seq4	ADCY2-like	Adenylatecyclase type 2-like (*Strongylocentrotus purpuratus*)	Up	4.18	XP_780688.3	75
**Lysosome**							
A4	comp74062_c0_seq5	NEU1-like	Sialidase-1 (*Strongylocentrotus purpuratus*)	Down	−1.88	DAA35227.1	85
A5	com78701_c0_seq2	AP-1-like	AP-1 complex subunit mu-1-like (*Strongylocentrotus purpuratus*)	Down	−2.67	XP_789616.3	77
S3	comp78293_c0_seq2	ABCA2-like	ATP-binding cassette sub-family A member 2-like (*Cricetulus griseus*)	Up	2.49	XP_003514719.1	70
S4	comp78293_c0_seq4	ABCA2-like	ATP-binding cassette sub-family A member 2-like (*Cricetulus griseus*)	Up	2.49	XP_003514719.1	70
S5	comp79570_c0_seq6	SGSH-like	*N*-sulphoglucosamine sulphohydrolase-like (*Strongylocentrotus purpuratus*)	Up	1.01	XP_794467.1	70
S6	comp77223_c0_seq3	ABCA2-like	ATP-binding cassette sub-family A member 2, partial (*Strongylocentrotus purpuratus*)	Up	1.77	XP_798273.3	68
S7	comp80153_c0_seq15	AP-3-like	Adaptor-related protein complex 3, δ 1 subunit-like (*Strongylocentrotus purpuratus*)	Up	1.79	XP_002733668.1	69
S8	comp78750_c3_seq11	DNase-II like	Plancitoxin-1 (*Capitella teleta*)	Up	1.89	ELU06802.1	75
**Endocytosis**							
A6	comp76401_c0_seq2	VPS37-like	ESCRT-I complex subunit VPS37 (*Nematostella vectensis*)	Down	−3.6	XP_001624048.1	82
S9	comp77471_c1_seq34	rabaptin5-like	RabGTPase -binding effector protein 1-like (*Strongylocentrotus purpuratus*)	Up	1.8	XP_789966.3	77
S10	comp80156_c1_seq5	AP-2-like	AP-2 complex subunit alpha-2 (*Rattus norvegicus*)	Up	1.16	NP_112270.2	74
S11	comp77877_c0_seq1	CHMP5-like	Charged multivesicular body protein 5-like (*Strongylocentrotus purpuratus*)	Up	1.76	XP_786663.1	72
S12	comp75233_c0_seq13	PAR6-like	partitioning defective 6 (*Hemicentrotus pulcherrimus*)	Down	−2.11	BAF99001.1	77
S13	comp79698_c0_seq6	EGFR/ RTK-like	Epidermal growth factor receptor (*Apostichopus japonicas*)	Up	1.32	AEY55412.1	97
**ERBB signaling pathway**							
A7	comp76122_c1_seq21	NCK2-like	Cytoplasmic protein NCK2 (*Strongylocentrotus purpuratus*)	Up	3.62	XP_784072.1	72
A8	comp76122_c1_seq7	NCK2-like	Cytoplasmic protein NCK2 (*Strongylocentrotus purpuratus*)	Up	2.72	XP_784072.2	72
A3	comp79708_c0_seq1	STAT5B-like	Signal transducer and activator of transcription 5B (*Strongylocentrotus purpuratus*)	Down	−3.81	XP_003723422.1	70
S13	comp79698_c0_seq6	EGFR/RTK-like	Epidermal growth factor receptor (*Apostichopus japonicas*)	Up	1.32	AEY55412.1	97
**MAPK signaling pathway**							
A9	comp77146_c0_seq3	MAP3K4-like	Mitogen-activated protein kinase kinase kinase 4 (*Strongylocentrotus purpuratus*)	Down	−2.23	XP_784029.3	72
A10	comp78357_c1_seq8	MAPK10-like	Mitogen-activated protein kinase 10 (*Strongylocentrotus purpuratus*)	Down	−4.43	XP_786040.3	75
S1	comp79328_c1_seq13	NFKB-like	Nuclear factor NF-κB p105 subunit (*Apostichopus japonicas*)	Up	1.8	AEP33644.1	68
S13	comp79698_c0_seq6	EGFR/RTK-like	Epidermal growth factor receptor (*Apostichopus japonicas*)	Up	1.32	AEY55412.1	97
S14	comp80408_c0_seq17	FLNA-like	Filamin-A (*Strongylocentrotus purpuratus*)	Up	1.75	XP_792145.3	74

A, represents disease-resistant gene; S, represents susceptibility gene. Log_2_ FC (fold change) indicates differential expression level of disease-resistant group (A) relative to the control group (K). “−”, indicates fold change of down-regulation.

**Table 5:** Other putative disease-resistant genes.

Coding Number	Contig ID	Gene Name	Predict Function	Regulation	Log_2_ FC	Accession Number in Nr Database	Identities (%)
A11	comp71589_c0_seq4	COX19-like	cytochrome c oxidase assembly protein COX19 (*Danio rerio*)	Down	−2.64	NP_001104010.1	72
A12	comp72396_c0_seq2	DDX47-like	probable ATP-dependent RNA helicase DDX47-like (*Strongylocentrotus purpuratus*)	Down	−4.9	XP_786173.3	76
A13	comp72841_c2_seq2	Trmt1-like	tRNA methyltransferase 1-like (*Saccoglossus kowalevskii*)	Down	−3.12	XP_002736321.1	67
A14	comp73256_c0_seq3	Hrsp12-like	heat-responsive protein 12 (*Mus musculus*)	Down	−3.8	EDL08846.1	77
A15	comp74533_c0_seq6	CNOT10-like	CCR4-NOT transcription complex subunit 10-like (*Ornithorhynchus anatinus*)	Down	−2.52	XP_001509062.1	76
A16	comp74754_c1_seq1	phyhd1-like	phytanoyl-CoA dioxygenase domain-containing protein 1-like (*Strongylocentrotus purpuratus*)	Down	−3.55	XP_789562.2	69
A17	comp74908_c0_seq5	ehhadh-like	Peroxisomal bifunctional enzyme (*Branchiostoma floridae*)	Down	−3.86	XP_002593843.1	73
A18	comp75055_c2_seq2	DHX35-like	probable ATP-dependent RNA helicase DHX35-like (*Strongylocentrotus purpuratus*)	Down	−3.6	XP_783015.1	66
A19	comp75531_c0_seq3	RIOK3-like	Serine/threonine-protein kinase RIO3 (*Saccoglossus kowalevskii*)	Down	−2.19	XP_002736242.1	69
A20	comp76071_c1_seq12	Map2k6-like	Dual specificity mitogen-activated protein kinase kinase 6 (*Capitella teleta*)	Down	−2.71	ELT91393.1	72
A21	comp76305_c0_seq6	Gvin1-like	interferon-induced very large GTPase 1-like isoform X2 (*Danio rerio*)	Down	−2.67	XP_684086.4	83
A22	comp76655_c1_seq14	Ndufb3-like	NADH dehydrogenase (ubiquinone) 1 β subcomplex subunit 3-like (*Strongylocentrotus purpuratus*)	Down	−1.02	XP_783578.1	81
A23	comp76725_c0_seq4	PRPFF19-like	pre-mRNA-processing factor 19 (*Strongylocentrotus purpuratus*)	Down	−2.97	XP_787949.3	74
A24	comp77143_c0_seq19	Mapkap1-like	target of rapamycin complex 2 subunit MAPKAP1-like (*Strongylocentrotus purpuratus*)	Down	−7.52	XP_787234.2	65
A25	comp77913_c0_seq1	V1g163483-like	Inosine triphosphate pyrophosphatase (*Rana catesbeiana*)	Down	−3.36	ACO51724.1	75
A26	comp78256_c0_seq1	SMU1-like	WD40 repeat-containing protein SMU1 (*Gallus gallus*)	Down	−2.46	NP_001007980.1	76
A27	comp78900_c0_seq70	ND5-like	NADH dehydrogenase subunit 5 (*Apostichopus japonicas*)	Up	1.15	YP_002836162.1	100
A28	comp79236_c0_seq23	YPEL5-like	protein yippee-like 5-like isoform 2 (*Strongylocentrotus purpuratus*)	Down	−3.51	XP_786314.1	75
A29	comp80082_c0_seq9	Usp39-like	tri-snRNP-associated protein 2 (*Strongylocentrotus purpuratus*)	Down	−4.33	XP_001185686.2	71
A30	comp80196_c0_seq6	Hsp70Ab-like	heat shock protein 70 (*Apostichopus japonicas*)	Up	4.6	ACJ54702.1	75

Log_2_ FC (fold change) indicates differential expression level of susceptibility group (S) relative to the control group (K). “−”, indicates fold change of down-regulation.

**Table 6:** Other putative susceptibility genes.

Coding Number	Contig ID	Gene Name	Predict Function	Regulation	Log_2_ FC	Accession Number in Nr Database	Identities (%)
S15	comp73644_c0_seq2	ARHGAP39-like	Rho GTPase-activating protein 39 (*Capitella teleta*)	Up	3.14	ELT94447.1	66
S16	comp74218_c0_seq25	ftsjd2-like	cap-specific mRNA (nucleoside-2'-*O*-)-methyltransferase 1-like (*Danio rerio*)	Up	2.04	XP_003729301.1	70
S17	comp75066_c0_seq3	DMBT1-like	scavenger receptor cysteine-rich protein type 12 precursor (*Strongylocentrotus purpuratus*)	Up	1.38	NP_999762.1	70
S18	comp73655_c0_seq9	Calr-like	Calreticulin (*Strongylocentrotus purpuratus*)	Up	1.33	XM_006792233.1	77
S19	comp72192_c0_seq1	ATG5-like	autophagy-related protein 5 (*Strongylocentrotus purpuratus*)	Up	1.32	XM_011665174.1	70

Log_2_ FC (fold change) indicates differential expression level of susceptibility group (S) relative to the control group (K). “−”, indicates fold change of down-regulation.

### 2.6. Immune Signaling Pathway

#### 2.6.1. MAPK Signaling Pathway

**Figure 10:**
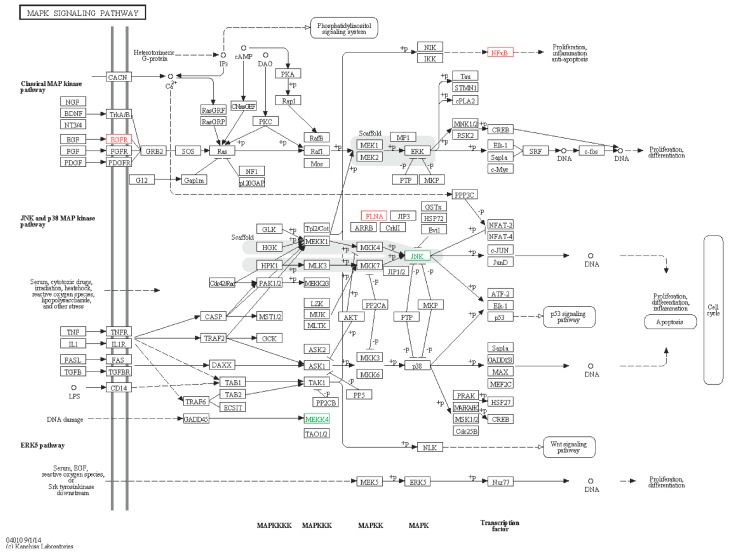
MAPK signaling pathway. Red boxes represent up-regulated genes, and green boxes represent down-regulated genes.

Five immune genes were enriched in the mitogen-activated protein kinase (MAPK) pathway, including JNK-like, MEKK4-like, NFkB-like, FLNA-like and EGFR-like (Figure 10). The MAPK cascade is a highly conserved module that is involved in various cellular functions, such as cell proliferation, differentiation and migration.

The MAPK signaling pathway exists widely in all eukaryotes from yeast to human. The genes involved in the MAPK pathway in other species have been identified including *Eriocheir sinensis* [7], *Pseudosciaena crocea* [41], but not in sea cucumber. The genes found in sea cucumber: EGFR, JNK, MEKK4, NF-κB and FLNA, had been identified in *Eriocheir sinensis*, but not in *Pseudosciaena crocea*. EGFR exists on the cell surface and is activated by binding with its specific ligands, including epidermal growth factor and transforming growth factor α (TGFα), and it activates several signaling cascades to convert extracellular cues into appropriate cellular responses, principally the MAPK, protein kinase B (Akt) and Jun N-terminal kinase (JNK) pathways, leading to DNA synthesis and cell proliferation [42]. FLNA gene serves as a scaffold for a wide range of cytoplasmic signaling proteins. Moreover, the lack of filamin A damages cyclinB relevant proteins, and consequently delays the initiation and progression of mitosis [43]. MEKK4 is a member of MAPK family and could activate downstream MAPK kinase [44]. The c-Jun N-terminal kinases (JNKs) belonging to a large group of serine/threonine (Ser/Thr) protein kinase from the MAPK family, could phosphorylate and activate the transcription factor c-Jun [45]. NF-kappa-B gene is a transcription factor, regulating expression of a number of genes that participate in the inflammatory response, immune response, cell growth and apoptosis [46]. NF-κB p105 plays a role in the activation of NF-κB as a MAPK kinase signaling regulatory protein [47].

#### 2.6.2. ERBB Signaling Pathway

**Figure 11:**
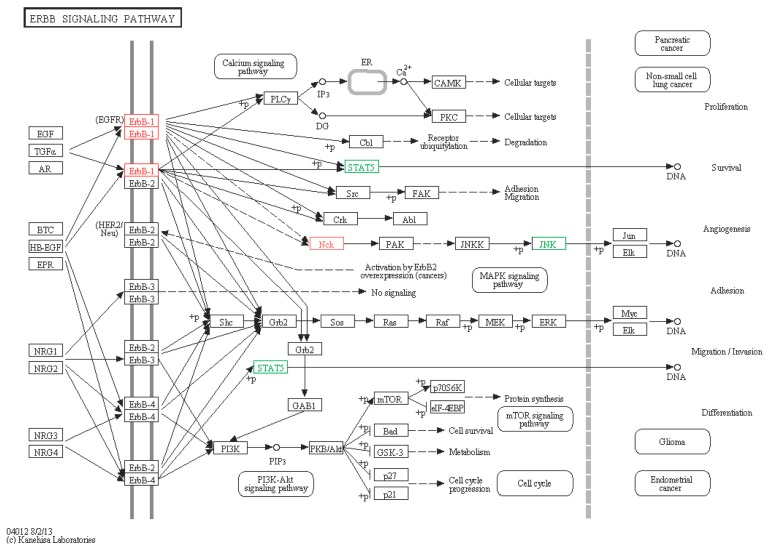
ERBB signaling pathway. Red boxes represent up-regulated genes, and green boxes represent down-regulated genes.

Four genes of sea cucumber were enriched in the ERBB pathway on the basis of our analysis (Figure 11), including ERBB-1-like (EFGR), STAT5-like, JNK-like, and Nck-like. The ERBB family of receptor tyrosine kinases (RTKs) couples binding of extracellular growth factor ligands to intracellular signaling pathways and regulates diverse biologic responses, including proliferation, differentiation, cell motility, and survival.

The ERBB pathway has been investigated widely in human, however, few studies have been done in other animal species, including sea cucumber. STAT5 is phosphorylated by EGFR stimulation and binds with specific DNA, further activating or inactivating transcription [48,49]. Nck regulates cell cycle arrest after DNA damage in some pathways including a translocation of Nck to the nucleus [50].

#### 2.6.3. Lysosomes

**Figure 12:**
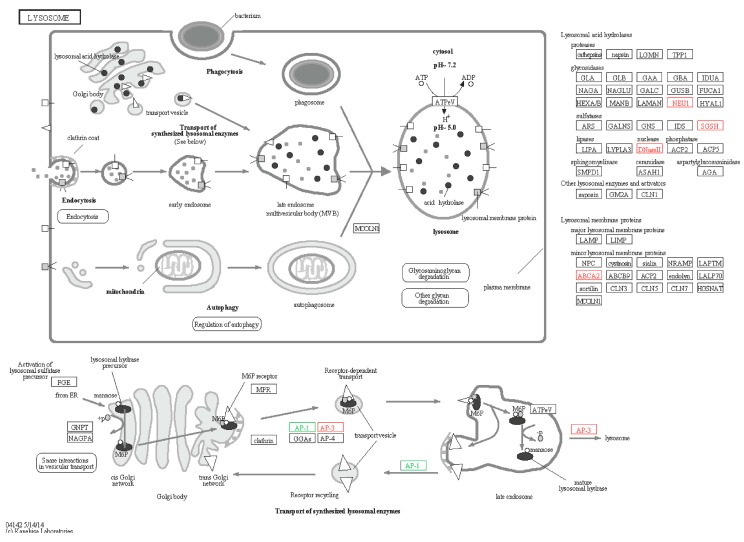
Lysosome signaling pathway. Red boxes represent up-regulated genes, and green boxes represent down-regulated genes.

The hydrolysis of lysosomes is an important process of immune response for sea cucumber, which degrades pathogens through hydrolytic enzymes. The acidic hydrolytic enzymes of lysosomes include protease, glycosidase, sulfatase and lipase. Lysosomes include some membrane proteins such as LAMP, LIMP, and ABCA2. SGSH-like, DNaseII-like, ABCA2-like, AP-3-like, NEU1-like and AP-1-like (Figure 12) involved in lysosome were identified, among which NEU1-like belongs to glycosidase, SGSH-like belongs to sulfatase, DNaseII-like belongs to nuclease, ABCA2-like belongs to membrane proteins, and AP-1-like and AP-3-like are members of the clathrin family. The lysosome produces various acidic hydrolysis after *Vibrio splendidus* infection, therefore SGSH-like, DNaseII-like and NEU1-like could represent up-regulated expression. ABCA2-like may be relevant to transmembrane transport, hence, foreign pathogens are transported into the cell via membrane proteins and further hydrolysis [51]. AP-1 is mainly localized to the trans-Golgi network (TGN) and mediates protein trafficking between the TGN and endosomes [52,53]. AP-3 localized to endosomes and/or the TGN and in mammals, is thought to mediate protein sorting to lysosomes and specialized endosomal-lysosomal organelles [54,55]. Therefore, we inferred that AP-1-like and AP-3-like also change in response to the infection of *Vibrio splendidus.*

#### 2.6.4. Endocytosis

**Figure 13:**
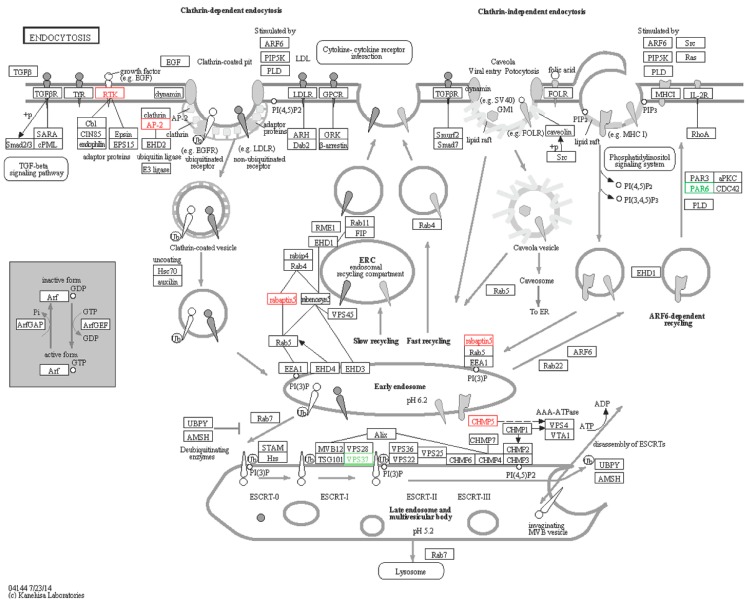
Endocytosis signaling pathway. Red boxes represent up-regulated genes, and green boxes represent down-regulated genes.

Endocytosis is a process which brings extracellular large molecules or other cells (like bacteria) into the cell interior by cytomorphosis. There are three types of endocytosis: phagocytosis, pinocytosis and receptor mediated endocytosis. RTK/EGFR-like, AP-2-like, PAR6-like, rabaptin5-like, CHMP5-like, and VPS37-like (Figure 13) were the DEGs enriched in endocytosis, among which VPS37-like and PAR6-like were down-regulated. AP-2 is localized to the plasma membrane and mediates receptor endocytosis [56,57,58]. CHMP5 participated in biosynthesis of multivesicular bodies in endocytosis [59], therefore its expression will be up-regulated after *Vibrio* *splendidus* infection. Depletion of HCRP1/hVps37A in mammals hinders degradation of EGFR [60]. Rabaptin 5 is a major component of clathrin-coated vesicles in the Golgi network, taking part in endosomal recycling compartment with Rab4 [61]. PAR6 is also a key adaptor that links Cdc42 and atypical PKCs to Par3 in endocytosis [62].

#### 2.6.5. Chemokine Signaling Pathway

**Figure 14:**
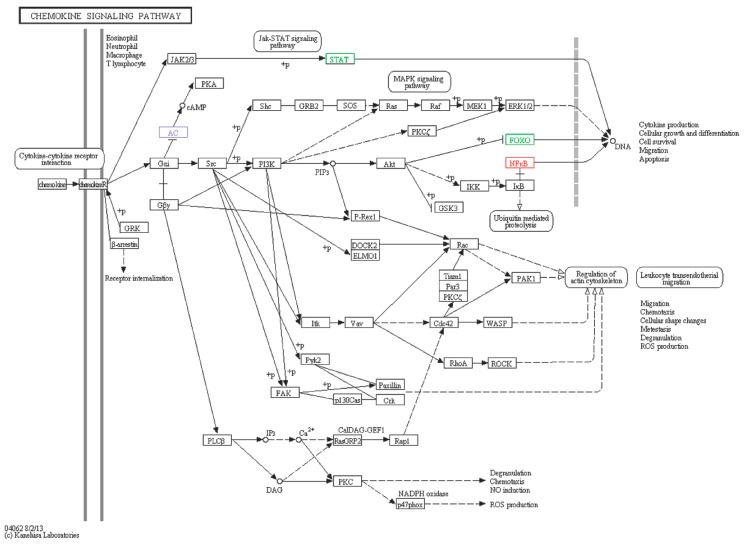
Chemokine signaling pathway. Red boxes represent up-regulated genes, green boxes represent down-regulated genes, and blue box includes two genes of which one is an up-regulated gene and the other is a down-regulated gene.

The chemokine receptor is one kind of small protein polypeptide of the cytokines superfamily and contains specific Cysteine motifs in their amino acid sequence [63]. Chemokines and their receptors are also important factors in B and T cell development [64,65], infections, angiogenesis, and tumor growth as well as metastasis. AC-like, NFkB-like, FOXO-like and STAT5B-like (Figure 14) participated in chemokine signaling pathways, of which FOXO-like and STAT-like were down-regulated. We predicted that the pathways including FOXO and STAT were inhibited, and immune response was depended mainly on the pathways involving in NFkB. Although above-mentioned genes are involved in immune signaling pathways, it is very necessary to validate their functions further.

### 2.7. Differential Expression Verification of Putative Genes

**Figure 15:**
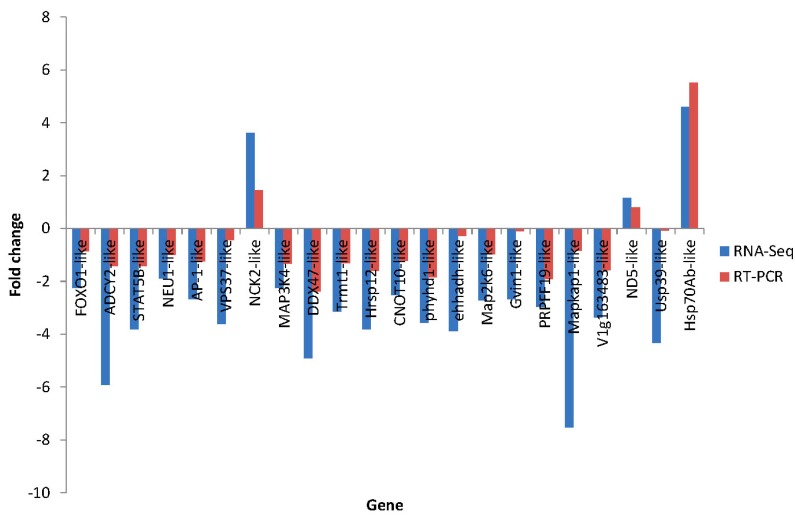
Comparison of 22 putative disease-resistant gene expression levels between RNA-Seq (blue bar) and RT-PCR (red bar). “−”indicates down-regulation.

**Figure 16:**
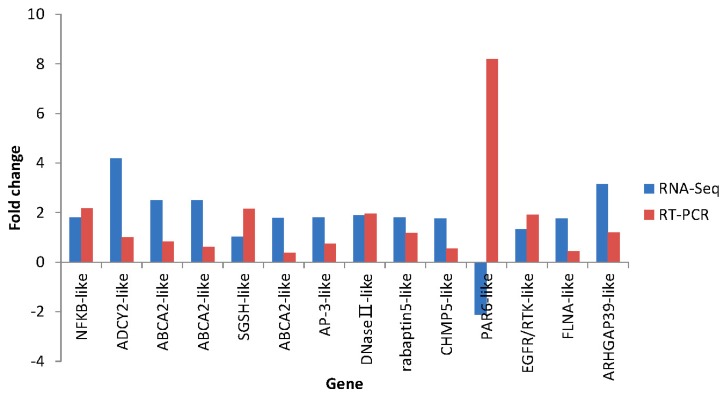
Comparison of 14 putative susceptibility gene expression levels between RNA-Seq (blue bar) and RT-PCR (red bar). “−”indicates down-regulation.

The primers of 36 genes were designed and all primer sequences are listed in Table S1. The candidate immune genes were used for qRT-PCR validation. The results showed the differential expression level of these genes between the control group and the disease-resistant group or susceptibility group (Figure 15 and Figure 16). Among them, up-regulation or down-regulation of 35 genes are consistent with the results of RNA-Seq. In conclusion, validation results (qRT-PCR *vs.* RNA-Seq) are overall reliable with the clear exception of PAR6-like, but further studies are still needed to verify the functions of these genes.

## 3. Experimental Section

### 3.1. Sea Cucumber and Microbial Challenge

Two hundred healthy sea cucumber juveniles from a culture corporation in Qingdao with average weight of 10.3 ± 2.1 g were randomly selected from a full-sib family which is medium-sensitive to *Vibrio splendidus* and acclimatized in 4 plastic tanks (83 cm × 64 cm × 60 cm) with filtered ozone sterilized seawater for a week. The temperature was maintained at 15 ± 1.5 °C during the whole period of the experiment. The water was changed every three days and the sea cucumbers of treatment group were fed with formula every three days after changing the water.

*Vibrio splendidus* strain used in the experiment was initially isolated from a skin ulceration syndrome sea cucumber and identified as the pathogen of this syndrome in our laboratory [6]. Cryopreserved *Vibrio splendidus* strain were revived on tryptone soy broth medium (TSB) medium, and then inoculated into liquid TSB medium at 28 °C for 12 h with shaking at 200 rpm. The cultured bacteria were collected by centrifuging at 4000 rpm for 2 min, and then re-suspended in sterilized seawater.

For microbial challenge, 1 tank with 50 samples was served as control (without any treatment), and the other 3 tanks with 150 samples were immersed with high density of *Vibrio splendidus* at the final pathogen concentration of 10^8^ cfu·mL^−1^. The challenge experiment lasted for 30 days and the water was changed every three days. The onset of skin ulceration syndrome was observed from 3 to 25 days in succession after the challenge. The skin ulceration syndrome appeared in 96 sea cucumbers (64% of the challenged sea cucumbers) and the residual 54 sea cucumbers (36%) were not infected by the disease. The first 25% infected sea cucumbers (24 sea cucumbers) were considered as bacterial-susceptible group (S) and the 36 uninfected sea cucumbers were considered as bacterial-resistant group (A). In all, three groups: control group (K), bacterial-resistant group (A) and bacterial-susceptible group (S) were used for the next steps.

### 3.2. Total RNA Extraction and cDNA Library Construction

Three sea cucumber individuals from each group were collected (S1, S2, and S3 from bacterial-susceptible group with obvious symptoms, A1, A2 and A3from bacterial-resistant group, K1 K2 and K3 from the control group). Coelomic fluid weas extracted and centrifuged at 3000 rpm for 2 min to harvest the coelomocytes. Total RNA was extracted using TaKaRa Mini BEST Universal RNA Extract Kit (Takara, Dalian, China). Total RNA quantity and purity were analyzed using Bioanalyzer 2100 and RNA 6000 Nano Lab Chip Kit (Agilent, Palo Alto, CA, USA) with RIN number >7.0 [66,67]. Nine RNA samples were collected from the 9 samples. RNA-Seq of every sample was performed respectively for gene expression profile analysis. Approximately 10 μg of total coelomocyte RNA was subjected to isolate Poly (A) mRNA with poly-T oligo attached magnetic beads (Invitrogen, Carlsbad, CA, USA). Following purification, the mRNA is fragmented into small pieces using divalent cations under elevated temperature. Then the cleaved RNA fragments were reverse-transcribed to create the final cDNA library in accordance with the protocol of the mRNA-Seq sample preparation kit (Illumina, San Diego, CA, USA), and the average insert size for the paired-end libraries was 300 bp (±50 bp). Subsequently, we performed the paired-end sequencing on an Illumina Hiseq2500 (LC Sciences, Hongzhou, China), following the recommended protocol from the vendor.

### 3.3. Sequencing and Assembly

Transcriptome sequence of the pooled nine samples was conducted using the Illumina paired-end RNA-Seq approach with Illumina 2500 sequence platform. Prior to assembly, the raw reads were first filtered by removing the adapter sequences, primer sequences and potential contaminations, which are the reads with unknown base greater than 5 and also with low-quality (<Q20) with existing tools: CutAdapt, NGS QC Toolkit and Trimmomatic. After that, paired-end trimmed reads were produced. The raw sequence data were then submitted to the NCBI Short Read Archive with accession number of SRP 057956. Also, trimmed reads were assembled using Trinity (Available online: http://trinityrnaseq.sourceforge.net/) and to remove the effect of different isoforms or alternative splicing, the longest contig of each isoform set was selected as the representative contig in the downstream analysis.

### 3.4. Annotation of Representative Contig

Representative contigs were first annotated to protein databases (download date: 7 March 2014) Nr, Swiss-prot, Pfam, KEGG and COG separately. Gene names were assigned to each assembled sequence based on the best BLAST hit (highest score). For homologous annotation, sample representative contigs were compared with NCBI non-redundant protein (Nr) (Available online: ftp://ftp.ncbi.nlm.nih.gov/blast/db/FASTA/nr.gz), Swiss-Prot (Available online: ftp://ftp.uniprot.org/pub/databases/uniprot/current_release/knowledgebase/complete/uniprot_sprot.fasta.gz), Cluster of Orthologous Groups (COG) (Available online: http://www.ncbi.nlm.nih.gov/COG/grace/shokog.cgi), Kyoto Encyclopedia of Genes and Genomes (KEGG) (Available online: http://www.kegg.jp/kegg/download/) and Pfam (Available online: ftp://ftp.sanger.ac.uk/pub/databases/Pfam/releases/Pfam27.0/Pfam-A.fasta.gz) database using algorithm blastx with *E*-value cut-off of 10^−5^. Gene ontology (GO) categories [68] were used for gene annotation using the BLAST 2 GO software [69,70]. The top 20 hits extracted from the blastx results were used for gene annotation and GO analysis (level 2), illustrating general functional categories. KEGG pathways were assigned to the assembled sequences using the online KEGG Automatic Annotation Server (KAAS, available online: http://www.genome. jp/kegg/kaas/). The bi-directional best hit (BBH) method was used to obtain KEGG Orthology (KO) assignment [71].

### 3.5. SNPs and SSRs Detection

The SNPs in the transcriptome level were analyzed based on the massively parallel Illumina technology. The Bowtie (Available online: http://bowtie-bio.sourceforge.net/) and Samtools (Available online: http://samtools.sourceforge.net/) software with default parameters (cDNA mode) were used to identify the SNPs. The SNP identification was limited to the transcripts (≥200 bp) containing at least 100 reads for each allele. The sample data were mapped to the contig via the Bowtie software after pretreatment, based on the library of transcription. Further SNP analysis was done according to the mapping results, and then variable sites with higher possibility were further filtered using the software of Samtools.

The MISA (Microsatellite) Perl script (Available online: http://pgrc.ipkgatersleben.de/misa) was used for the identification of SSRs. The BatchPrimer3 V1.0 program was used to design primers pairs for amplification of the SSR motifs [72]. Monomers, Dimers, Trimers, Quadmers, Pentamers and Hexamers were all considered as the searching criteria for SSRs in MISA script.

### 3.6. Identification of Differentially Expressed Genes (DEGs)

To investigate the expression level of each representative contig in different groups, digital gene expression profiles of the three groups were constructed and transcripts expression levels were calculated using RPKM (Reads per kilobase of exon model per million mapped reads) [73]. *p*-Value was used to identify the DEGs between two groups using chi-square test (2 × 2), and the significance threshold of the *p*-value in multiple tests was set based on the FDR (FDR ≤ 0.05). The fold changes (log_2_ (RERPKM/PERPKM)) were also estimated according to the normalized gene expression levels. Considering the above researches, “*p*-value < 0.01 and |log_2_fold change| ≥ 1 (FDR ≤ 0.05)” were set as the threshold. Putative *Vibrio splendidus*-resistant genes were screened through comparing the expression level of genes between disease-resistant group (A) and control group (K), and susceptibility genes were screened through comparing the expression difference between bacterial-susceptible group (S) and control group (K).

### 3.7. Identifying Potential Immune Genes and Pathway Analysis

The gene ontology (GO) was conducted for Functional classification of the putative disease resistant and susceptibility genes, and the pathway analysis was carried out by using KEGG.

### 3.8. Validation of Illumina Sequencing Results by qRT-PCR

Quantitative RT-PCR (qRT-PCR) was used to verify the expression level of putative immune genes that were identified in RNA-Seq analysis. Primers were designed using the Primer5 software and β-actin gene was used as the reference gene [74]. The qRT-PCR reactions were performed in a 20 mL volume composed of 2 μL of cDNA, 4 μM of each primer, and 10 mL Master mix, 2× conc (Roche, Penzberg, Germany) in the Eppendorf Real time PCR System. The thermal cycling program was 95 °C for 10 min, followed by 40 cycles of 95 °C for 10 s, 57 °C for 20 s and 72 °C for 30 s. Melting curve analysis was performed by the end of each PCR to confirm the PCR specificity. Three replications were used for each qRT-PCR validation. The relative expression of target genes was calculated using the 2^−∆∆*C*t^ method [75]. Differential expression level between control group and experimental group was determined using Log_2_ (A/K) or Log_2_ (S/K).

## 4. Conclusions

We conducted transcriptome sequencing and gene expression profile analysis of coelomocytes RNA in sea cucumber; 30 potential disease-resistant genes and 19 potential susceptibility genes were obtained, respectively, according to GO, KEGG, NCBI annotation and relevant published references. Furthermore, the genes were involved in immune signaling pathways, such as Endocytosis, Lysosome, MAPK, ERBB, and Chemokine, playing key roles in the interactive network of genes. Our study might provide useful information for future investigation of defense mechanism for *Vibrio splendidus* challenge.

## Supplementary Materials

Supplementary materials can be found at http://www.mdpi.com/1422-0067/16/07/16347/s1.
