# Supplementary material for: Transcriptome Analysis and Discovery of Genes Involved in Immune Pathways from Coelomocytes of Sea Cucumber (Apostichopus japonicus) after Vibrio splendidus Challenge
Source: Int J Mol Sci. 2015 Jul 17;16(7):16347–77. doi: 10.3390/ijms160716347 (PMC4519954; doi:10.3390/ijms160716347)
Supplement: Supplementary file 1 [file ijms-16-16347-s001.pdf]

Supplementary Information

Table S1. Genes and specific primers used for real-time PCR.

NO.	Accession	Gene Name		Primer Sequence	Tm (°C)	Product Length (bp)
-	-	β-actin	-F	5′-AGGTTATGCTCTTCCTCACGCTAT-3′	57.7	-
			-R	5′-TCGCTCGTTGCCGATGGT-3′	59.6	
A1	comp76725_c0_seq6	FOXO1	-F	5′-GAGGCAGGGCGAAGAAAA-3′	61.41	110
			-R	5′-CCCCAAAGAAAACGGAAGTG-3′	61.74	
A2	comp78415_c0_seq14	ADCY2	-F	5′-AGCTGCGTGGAGAAGATCAAA-3′	62.35	122
			-R	5′-GCAAACTCCGTCAATACACCAA-3′	62.16	
A3	comp79708_c0_seq1	STAT5B	-F	5′-AGAAACCAGATTTTCAGCGTCAG-3′	61.97	109
			-R	5′-CCTTGGCTTGGCTCTCACTT-3′	61.84	
A4	comp74062_c0_seq5	NEU1	-F	5′-GGAGTTACGACGGAGGTCAAA-3′	61.41	142
			-R	5′-ACTCTCTTGCTGGTGCTTGCT-3′	61.67	
A5	com78701_c0_seq2	AP-1	-F	5′-CACGGTCGGCTACGCTAAGT-3′	62.62	137
			-R	5′-TCCCTTCTTGTTCCTCTGCTTC-3′	62.1	
A6	comp76401_c0_seq2	VPS37	-F	5′-GCCTGTCGGCTTTTAATTTGTC-3′	62.11	97
			-R	5′-TTTTCTCTGGCGTTCTTGTTCTC-3′	62.07	
A7	comp76122_c1_seq21	NCK2	-F	5′-CCACAGATCGTTGAGCTTCATT-3′	61.56	126
			-R	5′-TCTCGGTTTTCATCCTCATAGTTTC-3′	61.91	
A9	comp77146_c0_seq3	MAP3K4	-F	5′-CCCCATTTTCCTGTTTCTTATCC-3′	61.97	82
			-R	5′-GGTCGGCTTGTGCTCTAATCT-3′	60.78	
A12	comp72396_c0_seq2	DDX47	-F	5′-ACTTTGACATACCGACGCACTC-3′	61.48	275
			-R	5′-ACTTCCACCTCTGCCTTTCTTCT-3′	61.94	
A13	comp72841_c2_seq2	Trmt1	-F	5′-GGTTATCAAGTTTCAGGCACTCAC-3′	61.25	119
			-R	5′-TTTTCTCTCTTCACAGGGTTTTCC-3′	62.07	
A14	comp73256_c0_seq3	Hrsp12	-F	5′-ATGGTAGAGGGCGGTATTGAG-3′	60.35	179
			-R	5′-GGGAACTTGTCCGTGAAATAGG-3′	61.92	
A15	comp74533_c0_seq6	CNOT10	-F	5′-TGGTCATTTATATGCAGCAGAGG-3′	61.34	116
			-R	5′-CAGGAGAGGCAGGGTAGATAACA-3′	61.83	

Table S1. *Cont.*

NO.	Accession	Gene Name		Primer Sequence	Tm (°C)	Product Length (bp)
A17	comp74908_c0_seq5	ehhadh	-F	5′-GGTTTGCTATGGGTGCCTTC-3′	61.76	96
			-R	5′-GCCCCTCCCTCGTGATAAA-3′	62.27	
A18	comp75055_c2_seq2	DHX35	-F	5′-TGCAAAGAAGTGACCTTGGAAC-3′	61.56	140
			-R	5′-GGCACCCAGAGCATAGAGGA-3′	62.65	
A20	comp76071_c1_seq12	Map2k6	-F	5′-TAACGCAGATGTCCATCACAAGA-3′	62.84	138
			-R	5′-GCCTCCGTCAGCAAATCAC-3′	61.79	
A21	comp76305_c0_seq6	Gvin1	-F	5′-CTAACTGTCGTTGGTGCTGTATTTT-3′	60.80	87
			-R	5′-CTGTTTCCCTTGGTTGATGTAGTT-3′	60.65	
A23	comp76725_c0_seq4	PRPFF19	-F	5′-GCAGAGCGTAAGAAACGAGGA-3′	61.93	92
			-R	5′-CCTGGATGAGAGGCTTTAGGTTT-3′	62.07	
A24	comp77143_c0_seq19	Mapkap1	-F	5′-ATCGGGAACCAGCATCAAAA-3′	62.66	222
			-R	5′-TCGTCCACTTCACCCTCCTC-3′	62.60	
A25	comp77913_c0_seq1	V1g163483	-F	5′-AACACCAGGTACAATCGTGGAA-3′	61.56	101
			-R	5′-TGGGCATCTCGGCAAAC-3′	61.82	
A27	comp78900_c0_seq70	ND5	-F	5′-TCTACTCGAACGGATGCGAAA-3′	63.04	228
			-R	5′-CCACGGATGAAGCCCAAA-3′	62.99	
A29	comp80082_c0_seq9	Usp39	-F	5′-AATGGTACGAGATACAAGACCTTCA-3′	60.29	135
			-R	5′-CTGTGCTGTCACCTGCGTTAG-3′	62.01	
A30	comp80196_c0_seq6	Hsp70Ab	-F	5′-GGGAATGGGATGGCGTTAG-3′	62.51	138
			-R	5′-CGATCCTGATGATAGTGACTTGTTG-3′	62.09	
S1	comp79328_c1_seq13	NFKB	-F	5′-TGGAGTTGCCTTCCCACA-3′	61.26	133
			-R	5′-GAAAAGCACCCGCCACA-3′	61.84	
S2	comp74502_c1_s	ADCY2	-F	5′-AAAAGTTTTCCAGACTTCCAGATCC-3′	62.21	83
			-R	5′-GCCCACGAGAATACACAGCA-3′	62.21	

Table S1. *Cont.*

NO.	Accession	Gene Name		Primer Sequence	Tm (°C)	Product Length (bp)
S3	comp78293_c0_seq2	ABCA2	-F	5′-ACCAGGAAGCTGGGAAAACA-3′	61.94	83
			-R	5′-ACCCCTAAAAGACCAAAGCACTC-3′	61.99	
S4	comp78293_c0_seq4	ABCA2	-F	5′-GAGTCCTGAACAACGAAACAGAAA-3′	61.86	149
			-R	5′-CACCCCTAAAAGACCAAAGCAC-3′	62.06	
S5	comp79570_c0_seq6	SGSH	-F	5′-TGAGGAGATGAAGAAGTCCCTGT-3′	61.52	102
			-R	5′-AAATCAGCACACCCATCCAA-3′	61.31	
S6	comp77223_c0_seq3	ABCA2	-F	5′-TATGACCGCTGCTCTGATGG-3′	62.31	148
			-R	5′-TCCAGGCTTTGCTCTGTGG-3′	62.52	
S7	comp80153_c0_seq15	AP-3	-F	5′-CAGCGATCAGTTTGCACGA-3′	62.18	143
			-R	5′-CCACTCTCTCCACCACTCCA-3′	61.29	
S8	comp78750_c3_seq11	DNase II	-F	5′-GAAGATAGTCAGCAGCGTGGAA-3′	61.81	104
			-R	5′-CTGTGGTTGATTTTGTTGGTGAG-3′	61.7	
S9	comp77471_c1_seq34	rabaptin5	-F	5′-AGATGGCAAACGATCAATTCCT-3′	62.04	141
			-R	5′-TCTCTTGTTCCTCTCGCTGTTG-3′	62	
S11	comp77877_c0_seq1	CHMP5	-F	5′-AGACGTTGAAAGATACCAAAACCAC-3′	61.75	145
			-R	5′-ACCTCGTCCGTCATTTCCA-3′	61.48	
S12	comp75233_c0_seq13	PAR6	-F	5′-GTCAAACCAGCCAATCAGAGAAA-3′	62.7	190
			-R	5′-GCCTGTCCTCGTGTGTGTTG-3′	62.81	
S13	comp79698_c0_seq6	EGFR/RTK	-F	5′-AACGGGGTTTGCGAGATG-3′	62.02	160
			-R	5′-TGGGAGGTCTGTGGATTGG-3′	61.93	
S14	comp80408_c0_seq17	FLNA	-F	5′-ACATACCGTCAGTGTCAAGAACAAG-3′	61.69	107
			-R	5′-CACCTGCGTGAACTTTGTGAG-3′	61.86	
S15	comp73644_c0_seq2	ARHGAP39	-F	5′-ACGACGGAAAACTTCTTTGAGG-3′	61.83	127
			-R	5′-CGATGTGCTTGCGGATGT-3′	61.88	
